# Preventive Dentistry

**Published:** 1903-09-15

**Authors:** C. M. Wright


### PREVENTIVE DENTISTRY.

BY C. M. WRIGHT, D.D.S.

Every dentist recognizes as basal truths that surgically-clean teeth cannot be attacked by caries; that normal gums, mucous membranes and oral secretions will hinder and resist the development of disease-producing agents; that frequent expert operations upon the surfaces of the teeth and gums are required in ninety percent of the American people at some period of their lives to maintain a physiological condition, and that it is the special business of the dental profession to supply these expert operations. But we must also acknowledge that as a profession, we have, notwithstanding our knowledge of and firm belief in these basal principles, neglected one branch of expert operating, namely, the frequent and thorough cleansing of the entire surfaces of all the teeth of our patients, which is logically first in importance in preventive dentistry.

In earnest devotion to constructive, reparative, or more brilliant mechanical efforts we have shirked the responsibility of the important work of thoroughly cleansing and polishing the teeth and massaging and caring for the surrounding softer tissues. We have satisfied our conscience with the chair talks and lectures to our patients, We have recommended specially-devised tooth brushes. floss silk, powders and mouth washes. We have occasionally distributed tracts on "How to Care for the Teeth," just as our confreres in Europe are trying to do by law. In short, we have shifted the care of the teeth and mouth as regards cleanliness from our own to our patients' shoulders. From the present outlook, with our bridge-work and gold caps, our contour fillings and porcelain inlays, and our devotion to mechanics of a high order, we shall—with the exception of here and there a man—continue to neglect this "minor operation," which we all know holds such a dignified and important place with regard to preventive dentistry and preventive medicine.

It was for these reasons, and because I felt unable or unwilling to change the whole course of my practice, and because I believed that many of my fellow practitioners were in the same condition, that I submitted to my friends of the Cincinnati Odontological Society a plan for the forming of a subspecialty in dentistry which should be devoted to the fundamental preventive operations of polishing the teeth and caring for the mouth, not as Dr. Hunter calls it, "dentally," but daintily. The plan was published in June, 1902, number of the *Dental Digest*, page 446, under the title of "A Subspecialty in Dentistry," and consisted of four propositions: "1. The practitioners of this separate and yet most important branch of dentistry are to be women of education and refinement, who are seeking a field for work of an honorable and useful kind and among people of culture. 2. The dental colleges are to offer opportunities for this partial and separate training, the course to consist of lectures on the anatomy of the teeth and gums, special pathology and physiology, and a special clinical training in prophylactic therapeutics. 3. Upon the completion of this special course, which shall require one session, or one year of study and practice under instruction in the college infirmary, and after presenting satisfactory evidence of proficiency in the polishing of teeth and caring for the mouth, the college shall grant a certificate of competence to the graduate of this course. 4. With this training and the dental college certificate, these women may be employed by dentists for this special work, or may practice the same in parlors of their own, or at the homes of patients, the dentists giving their influence and recommending the new specialists, just as physicians and surgeons recommend and insist upon the services of the trained nurse or the masseure." I further said, "I think every one of you will agree that there could be no more valuable service in oral hygiene than that which such a class of specialists give, . . . . *especially in conjunction with our surgical treatment of Riggs' disease.*"

Dr. Truman in an editorial generously criticises the plan and remarks that "this proposition is worthy of serious consideration." He then adds with significant prophesy: "It is certainly true that we are on the eve of a radical change in dental practice. Not that old methods will be given up, but that new ones will be added, not alone for the direct preservation of the teeth from caries, but in the treatment of the oral cavity, to prevent through this source contamination of the entire physical organism. It must be clear that if the intelligent portion of the community had this matter thoroughly explained to them, there would be a rapid appreciation of its importance and an extensive demand for the services of carefully-instructed women."

Dr. Truman expresses some fears of these partially-educated specialists being tempted to overstep their bounds and wander into the, to them, forbidden grounds of general dental practice. He also sees no parallel between the relation of the trained nurse in medicine to the physician and of these subspecialists to the dentist, but the fact that the partially-educated dental profession does not trespass on the private domains of the physician, and also that these women must in the beginning at least be largely dependent upon the recognition and recommendation of the dentist for their employment, seem to me to be a barrier against invasion and a protection against infringement. Then we are supposed to be controlled by State laws regulating practice, and a modification of these laws might be adopted that, while permitting such specialists to practice, would also control and limit them as we are controlled and limited.

Dr. O. N. Heise, of Cincinnati, in an excellent paper entitled "Dentistry as a Specialty of Medicine," which was published in the *Dental Digest* for February, 1902, significantly remarks that in cases of accident under anesthetics—heart failure, persistent syncope, etc.—the dentist immediately seeks the services of a physician, showing simply that he feels his limitations. So it seems to me would these women practitioners of this well-defined subspecialty gladly remain within the scope of their priviledes.

In a private letter on this subject by Dr. S. A. Hopkins, of Boston, I learned that others have been impressed with the same hope, and I beg leave to quote a few sentences giving his experiences in this direction: "I would say that the idea of having trained assistants has occurred to me, and some time ago I made an attempt to secure such assistance for my office. I met with opposition from the State Board of Dental Examiners, who frankly told me that they would look upon that as the practice of dentistry and should feel it their duty to prosecute any one who performed even that slight operation without having first passed the Board and secured the usual license. I went even further and secured an opinion from one of our best lawyers, which was to the effect that under the present laws of Massachusetts such work could not be done. Consequently, our first step in this direction must be a change in some of the laws governing the practice of dentistry. . . . I heartily approve of your suggestion and hope you will keep on advocating the same until we accomplish our purpose."—*Digest.*

